# Shining a Light on Some Fundamental Research Opportunities in Semiconductor Photoelectrochemistry

**DOI:** 10.1021/acscentsci.5c02044

**Published:** 2026-03-09

**Authors:** Paul J. L. Bean, Dominic Covelli, Xinyi Elaine Shen, Nathan S. Lewis

**Affiliations:** † Division of Chemistry and Chemical Engineering, 127-72, 210 Noyes Laboratory, 6469 California Institute of Technology, Pasadena, California 91125, United States; ‡ Beckman Institute, California Institute of Technology, Pasadena, California 91125, United States

## Abstract

Decades of research in semiconductor photoelectrochemistry have yielded a deep understanding of charge transfer, energetics, and stability at solid–liquid interfaces. Theoretical frameworks developed by Gerischer and contemporaries, together with extensive experimental validation, have clarified the key principles affecting the interfacial kinetics and energetics of semiconductor photoelectrodes. Nevertheless, significant opportunities remain for advances in fundamental understanding of semiconductor photoelectrochemistry. Exciting opportunities include exploiting advances in theory, synthesis, and instrumentation to determine the chemical identity and reactivity of surface states; exerting control of band-edge energetics through molecular-level surface modification processes; and systematically improving emerging photoelectrode protection strategies to enable long-term photoelectrode operation under both oxidative and reductive conditions. Advanced morphologies, such as nanowire and microwire arrays, present new pathways to combine efficient light absorption with effective charge collection and catalyst integration. Unique light–matter interactions during photoelectrochemical deposition of p-type semiconductors readily allow preparation at scale of complex 3D morphologies that are difficult, if not impossible, to access by other methods. Continued exploration of these avenues will expand the fundamental understanding of semiconductor–liquid interfaces and could additionally advance the realization of efficient, stable, and scalable systems for solar fuel generation and other emerging photoelectrochemical applications.

## Graphical abstract

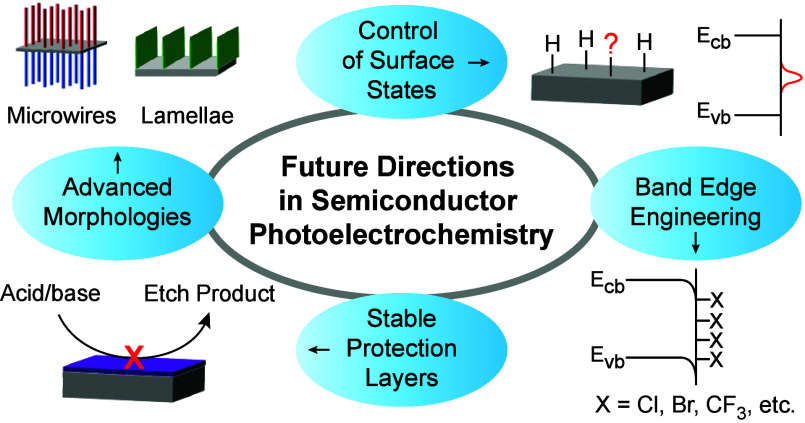

Semiconductor photoelectrodes have been the subject of fundamental and applied research for almost 3/4 of a century. Early studies of semiconductor–liquid junctions^1–4^ were performed in parallel with studies of solid-state Schottky barriers and p–n homojunctions.^5–7^ Semiconductor electrodes have a lower effective density of states than metals, and consequently produce an electric potential drop predominantly within the electrode as opposed to in the liquid part of the electric double layer.^8–12^ As opposed to metals that exhibit interfacial charge transfer only with one band of states (Figure 1a), semiconductor electrodes can undergo interfacial charge transfer with two energetically distinct bands of the electrode (Figure 1b).^11,13,14^ Theoretical expectations for the energetics and interfacial charge-transfer kinetics of semiconductor electrodes were therefore developed by modifying emerging classical and semiclassical theories for charge transfer across metal–liquid interfaces.^11,14–21^

**Figure 1:**
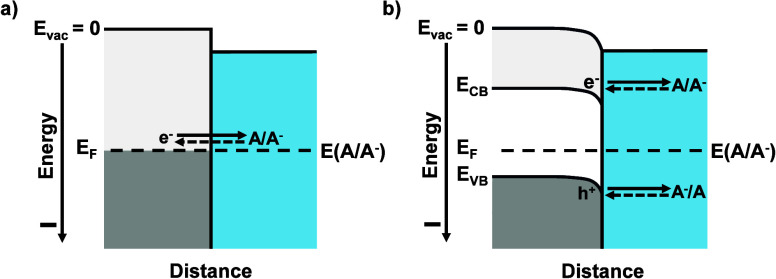
Energy-level diagrams of (a) a metal electrode exchanging electrons with a redox species in solution and (b) a semiconductor electrode, with two bands, exchanging charge with a redox couple in solution. All energy quantities are negative, i.e., bound, relative to the vacuum level. The electrochemical potential of the solution, **E**(A/A^–^), is equivalent to its Fermi level and at equilibrium is equal to the Fermi level of the electrode, **E**_F_. **E**_CB_ is the energy of the bottom of the conduction band and **E**_VB_ is the energy of the top of the valence band. In (a), charge transfer occurs from the filled and empty states in the metal near the Fermi level to the redox species in the solution. In (b), for a semiconductor, electrons exchange into and out of the solution at an energy near the bottom of the conduction band of the semiconductor, whereas holes exchange into and out of the solution at an energy near the top of the valence band of the semiconductor.

In the ensuing decades, most of the key aspects of these theoretical expectations for semiconductor photoelectrodes were verified experimentally. Initial measurements of aqueous interfacial charge-transfer rates on ZnO electrodes were confounded by the lack of a series of well-defined one-electron outer-sphere redox couples against which the theory could be evaluated analytically.^8,22^ Furthermore, measurements on small band gap semiconductors were confounded by corrosion as well as by changes in surface chemistry instigated by shifting electrode potentials.^8,11,23–25^ Subsequently, systems were painstakingly constructed with nonaqueous electrolytes, well-defined semiconductor surfaces, and minimal complications from ion adsorption, inner-sphere electron-transfer processes, and photocorrosion.^26–32^ Such systems showed the expected first-order kinetic rate law for charge transfer, and allowed determination of the rate constants for interfacial charge transfer from measurements of the current density as a function of electrode potential.^33–37^ Although the maximum rate constant for outer-sphere interfacial charge transfer was not quantified by Gerischer in the original formulation of the theory of semiconductor photoelectrochemistry,^11,38,39^ these measurements allowed for experimental determination of this key quantity and stimulated a variety of classical, semiclassical, and quantum mechanical treatments of the process.^10,17,18,21,34^ This approach also allowed identification of the Marcus inverted region,^35–37^ along with the predicted loss of image-charge stabilization that reduces the reorganization energy of a redox species near a metal electrode relative to a redox species near a semiconductor electrode.^36,40,41^ Well-defined surface functionalization processes, such as methylation of H-terminated Si(111) surfaces, moreover allowed measurement of the predicted band-edge shift upon introduction of a controlled dipole onto the electrode surface.^42–44^

Semiconductor photoelectrochemistry has also garnered much interest associated with potential practical applications. In the energy crisis of the 1970s, semiconductor photoelectrodes promised a simple, and thus potentially inexpensive, approach to converting solar energy directly into electricity and/or fuel.^45–49^ For context, by 1990, decades of work on inorganic photosensitizers to produce hydrogen and oxygen from sunlight had yielded partially complete chemical cycles with low quantum efficiencies in the near-UV part of the spectrum, exemplified by the photochemistry of species such as Ce^4+^(aq).^50,51^ A bridged Rh isocyanide dimer produced H_2_ upon irradiation with visible light in 5 M HCl­(aq), but the original Rh­(I) complex could not readily be regenerated from the oxidized Rh­(II)–Rh­(II) dimer by formation of O_2_(g).^52,53^ Various systems based on photosensitization of Ru­(bipy)_3_^2+^ and its derivatives have used sacrificial reagents to yield chemical reaction products such as oxidized amines but not to form a complete solar fuels cycle.^54–57^ Relatively slow oxidation of water to O_2_(g) by a blue Ru dimer was found to proceed with a limited turnover number before the complex decomposed.^58,59^ Moreover, in the 1970s, fabrication of solid-state junctions was technically difficult and expensive, with solar photovoltaic (PV) modules having a real cost per peak watt (W_p_) > 100 times the cost per W_p_ of PV modules today.^60–62^

Hence, when Fujishima and Honda showed in the early 1970s that TiO_2_ could produce H_2_ and O_2_ directly from sunlight,^45,63^ semiconductor photoelectrochemistry experienced an explosion of interest globally.^64,65^ A flurry of subsequent investigations showed that large band gap semiconductors readily formed H_2_ and O_2_ under sunlight.^66–69^ However, the large band gap of the functional, stable materials limits the efficiency and thus the potential energy-related application of such systems.^66,69,70^ Small band gap, potentially high-efficiency photoelectrodes are thermodynamically unstable for water splitting under illumination, and thus corrode.^25,71^ Particles of large band gap semiconductors, such as TiO_2_, with or without a series of cocatalysts, provided photocatalysts for toxic waste degradation^72–74^ but have not yielded a viable solar fuel technology due to instability, inefficiency, and/or safety considerations of coevolving stoichiometric streams of H_2_ and O_2_ in the presence of active catalysts for recombination of the gases.^75–77^

A collection of high efficiency (>10%) regenerative photoelectrochemical cells was developed by use of special stabilizing electrolytes^49,78–80^ or by the use nonaqueous solvents.^29,31,81^ However, stability was obtained only for regenerative cells that produced electricity (Figure 2a) and not chemical fuels (Figure 2b). Meanwhile, the ease and cost of making p–n junctions of common semiconductor materials like Si, GaAs and CdTe decreased dramatically, to the point where solar PV modules now cost < $0.50/W_p_.^62^ Moreover, the cost of making the metallurgical junction is a small fraction of the total solar cell cost,^82^ limiting the opportunity for semiconductor–liquid junctions to compete technologically solely for production of solar electricity.

**Figure 2:**
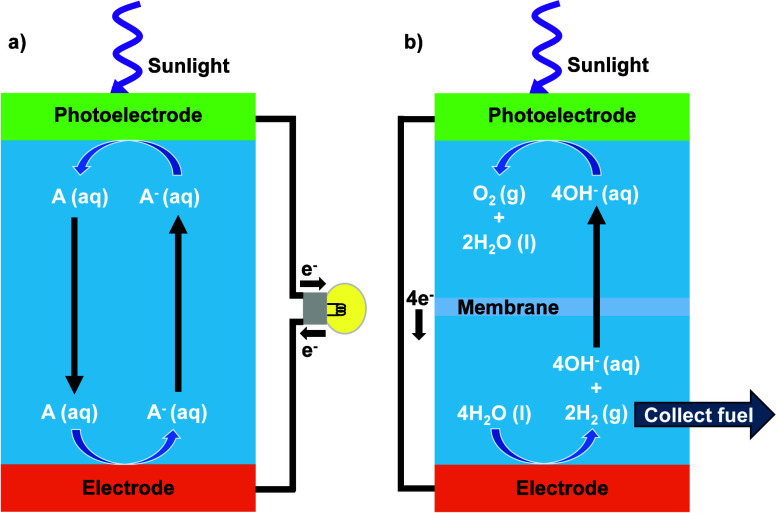
Diagrams demonstrating (a) a regenerative photoelectrochemical (PEC) cell generating usable electricity and (b) a fuel-producing PEC cell producing hydrogen. In a regenerative cell, the electrochemical half-reaction that occurs at the photoelectrode is identical to, but chemically the reverse of, the electrochemical half-reaction that occurs at the counter-electrode. Consequently, no net chemical change occurs in the cell and incident light is converted into an electrical current in the external circuit. In a fuel-forming cell, water is oxidized to O_2_(g) at the anode (in this example, the photoanode) and water is reduced to H_2_(g) and/or CO_2_ is reduced to CH_4_ or other reduced carbon species at the other electrode. For the example water-splitting cell shown in (b), to maintain electroneutrality and ensure no net chemical change in the electrolyte at steady state, except consumption of H_2_O to produce O_2_(g) and H_2_(g), protons must traverse the membrane in the same direction as movement of electrons in the external circuit, or hydroxide (OH^–^) anions must move across the membrane in a direction opposite to the flow of electrons. The membrane and/or separator is required to ensure robust separation of the gas streams, to prevent recombination and for safety reasons.

For over a half-century, buried metallurgical semiconductor junctions have been placed in contact with a liquid to produce electricity and/or fuel from sunlight.^83–91^ Relatively recent examples include the use of tandem III–V or triple-junction solar cells based on amorphous hydrogenated Si,^92^ in conjunction with inorganic or biological catalysts, to produce solar fuels.^93–97^ Such systems do not produce a space-charge region in the semiconductor due to the contact with the liquid electrolyte, and thus rigorously are not semiconductor photoelectrodes in either the fundamental or practical sense.^98,99^ Such liquid-immersed photovoltaic constructs have uniformly exhibited lower efficiencies and stabilities than if the photovoltaic cell were completely separated from the electrolyte and connected by an external circuit to the electrolytic cell.^85,87,100–105^

Given this state of development of semiconductor photoelectrochemistry, we evaluate some fundamental research opportunities that could be expeditiously advanced by leveraging recent progress in theory, surface chemistry, and analytical instrumentation. An extensive “Solar Fuels Roadmap” published in 2022 by over 40 authors contains nearly 200 references to recent work including applications of semiconductor photoelectrochemistry to solar fuels production.^106^ A recent review of activities in semiconductor photoelectrochemistry for solar fuels generation was authored by the director of the LiSA solar fuels hub.^107^ A very recent publication that has appeared after this Outlook was submitted, with the stated goal of presenting a roadmap for the next decade of research in semiconductor electrochemistry, by almost 70 authors, summarizes a broad collection of recent work and research directions, and contains a plethora of associated recent references.^108^ The focus of this Outlook is to complement these other recent “roadmaps” by critically highlighting barriers and fundamental research opportunities in a few selected areas with exceptionally high opportunities for imminent advancement, as opposed to contradicting, duplicating, or de-emphasizing by omission other fundamental and applied research efforts in the field.

In principle, the semiconductor–liquid interface affords an opportunity to obtain chemical control over both the interfacial energetics and interfacial charge-transfer kinetics. Bardeen elucidated the physics of surface states on semiconductor interfaces.^6^ Formation of strong bonds to the atoms that form surface traps should reduce the surface recombination velocity by reducing the number of midgap traps (Figure 3).^109–111^ A plethora of empirical treatments have been used for surface “passivation” both chemically and electrically,^112–115^ but identification of the actual chemical structure, bonding, and reactivity of surface states remains a frontier area. A complication is that the surfaces may have a combination of binding sites that, moreover, can undergo reconstruction during reaction. If the surface-state concentration of concern is small, the bound species are difficult to detect directly even by sensitive surface spectroscopic techniques.

**Figure 3:**
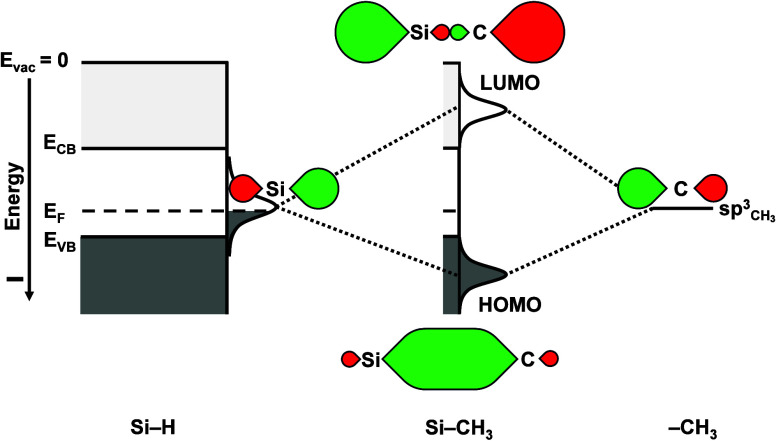
Molecular orbital diagram illustrating how formation of strong bonds to surface states can reduce the number of midgap traps at the surface of the semiconductor. For illustration, this example depicts the interaction between the C atom of a −CH_3_ fragment binding to an unterminated sp^3^ hybrid orbital on a Si surface atom. Bond formation will produce two molecular orbitals, one having a lower energy (and no nodes between the nuclei of the bonding atoms) than either of the original orbitals and one having a higher energy (and a nodal plane between the nuclei of the bonding atoms) than either of the original orbitals. States with energies in the middle of the band gap of the semiconductor are the most effective at recombining both electrons and holes, and consequently produce the highest surface recombination rate. Hence, strong bonds that shift the resulting orbital energies either higher or lower than midgap will reduce the rate of undesirable surface recombination.

One approach with a favorable outlook takes advantage of advances in theory and instrumentation by characterizing the surface states as a “chemical unknown”, i.e., deducing the identity of the surface sites of interest by elucidating their chemical reactivity.

Substantial progress has been made by treating the H-terminated Si(111) surface as a hindered silane, and using a variety of synthetic methods to obtain molecular-level control over the electrical, electronic, and electrochemical behavior of the resulting, chemically functionalized Si(111) surfaces.^42,116–120^ For example, alkylated Si(111) surfaces yield air-stable interfaces that provide nucleation sites for subsequent deposition of barrier films by atomic layer deposition;^121^ extend the stability of Si surfaces toward oxidation in a variety of ambients and electrolytes; and exhibit low surface recombination velocities in contact with air and electrolyte solutions under a variety of conditions.^122^ The methyl-terminated Si(111) surface is especially interesting because −CH_3_ groups can terminate every atop site on an unreconstructed Si(111) surface (Figure 4).^117,118,123–130^ Alkylation therefore shifts the Si band-edge positions and increases the photovoltage of n-Si(111) photoanodes without introducing deleterious Fermi-level pinning at the functionalized Si surface.^44^

**Figure 4:**
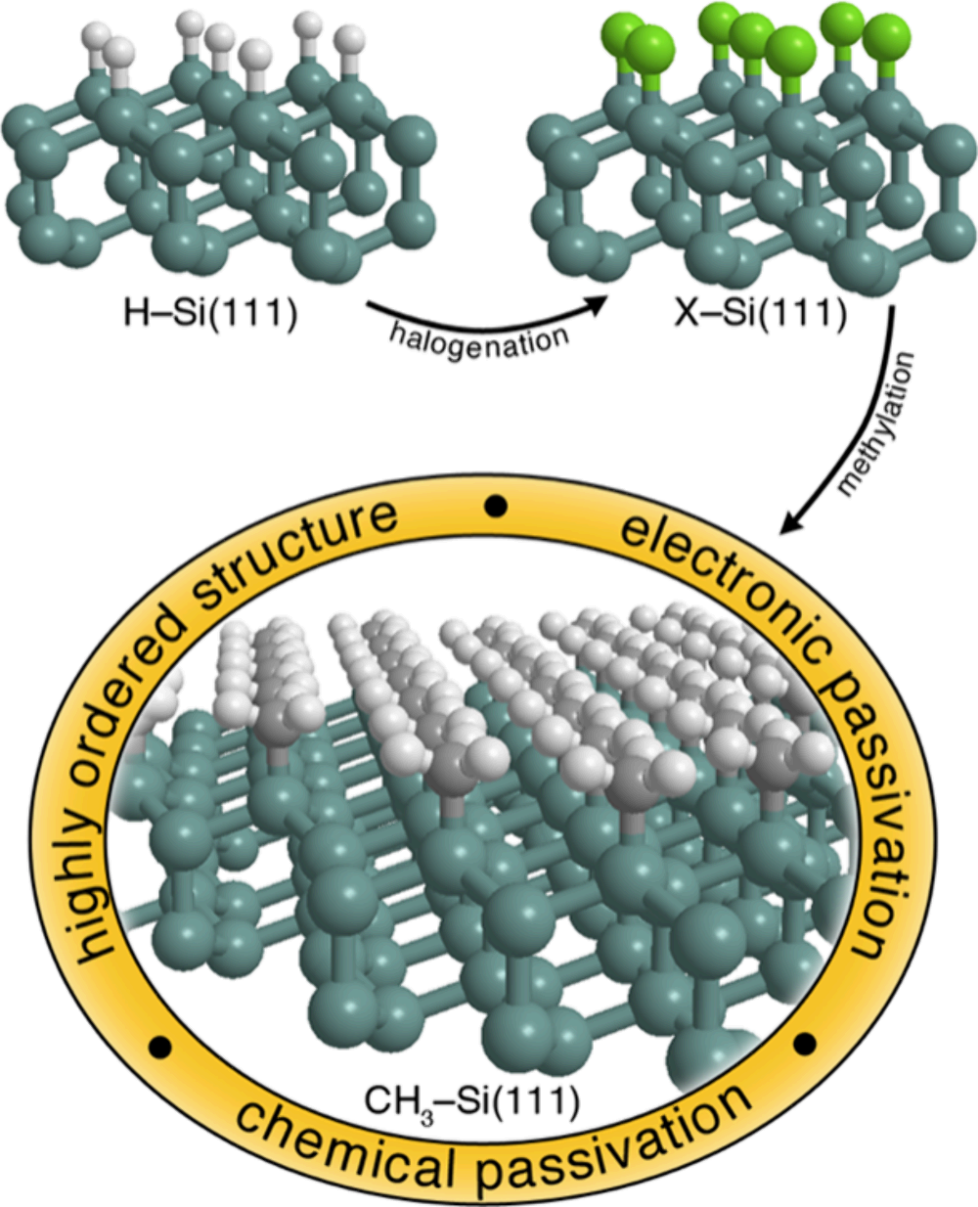
An unreconstructed H-terminated Si(111) surface has all of the Si–H bonds perpendicular to the surface plane, with every atop Si atom terminated by bonding between a Si sp^3^ hybrid orbital and a H 1s orbital. Chlorination with a mild chlorine radical source, such as PCl_5_, followed by reaction with a Grignard reagent, produces a methyl-terminated Si surface in which every Si atop atom is bonded to a methyl group. The lack of dangling bonds at the Si surface results in a low midgap trap density and produces a low surface recombination rate, and the inertness of the Si–C bonds inhibits reaction of the surface with air or water to form deleterious surface oxides. Reproduced from ref 43. Copyright 2014 American Chemical Society.

This chemistry has subsequently been extended to obtain molecular-level control over Ge(111) surfaces.^131–134^ Moreover, an analogous approach has been developed to elaborate the reaction chemistry of the polar faces of InP (Figure 5).^135–137^ The reaction chemistry, along with surface spectroscopy, clearly indicates that the surface functionalization process on the P-rich (111)B face of InP proceeds through P–OH bonds. In addition, the low resulting surface recombination velocities imply that less than 1 electrically active defect atom is present for every 10^5^ atoms on the functionalized InP surface. GaP(111)­A and GaP(111)­B faces have been differentially functionalized using analogous methods.^138–140^

**Figure 5:**
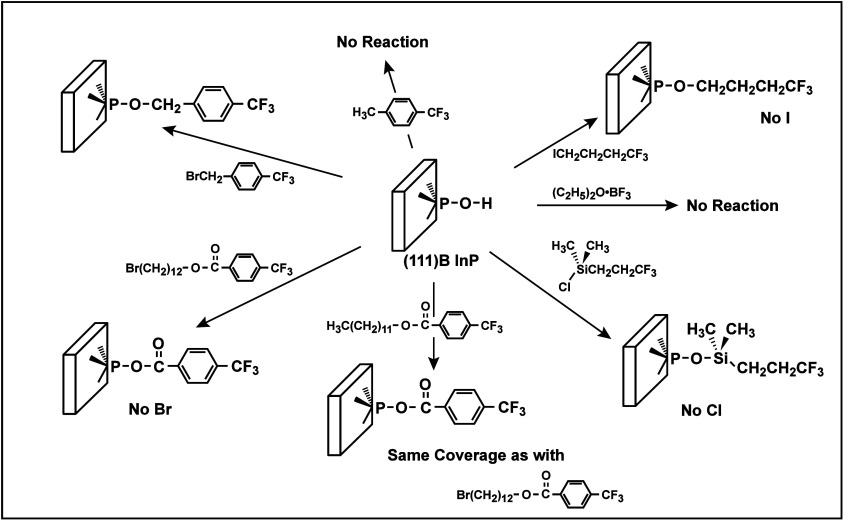
Summary of the reactivity of the (111)B face of InP with various reagents, as determined by infrared spectroscopy and X-ray photoelectron spectroscopy of the surface, before and after, respectively, exposure to various reagents. The −CF_3_ groups serve as “tags” to allow differentiation of the bound reagent from adventitious hydrocarbon species that might adsorb at various stages onto the surface, both by the presence of bound fluorine atoms and by the distinctive, readily detectable change in the X-ray photoelectron C 1s emission resulting from the presence of the highly electron-withdrawing F species bonded to the central tagged carbon atom. Reproduced from ref 135. Copyright 1999 American Chemical Society.

The outlook for developing methods of molecular-level control through surface chemistry is especially promising for 2-D layered materials such as MoSe_2_ and WSe_2_.^141^ Specifically, defect and corrosion sites could be spatially resolved onto edge versus terrace sites, and the different reaction sites could be identified further based on their different chemical reactivities, with precedent based on the improved photoelectrochemical performance of these surfaces after exposure to coordinating ligands such as phosphines or pyridine.^142^ Moreover, spatially resolved scanning probe techniques, as well as impedance spectroscopy combined with surface spectroscopy methods such as scanning Auger with unique element tags on the reactive groups attached to the surface, and additionally possibly time-resolved probes of lateral energy and electron transfer processes at semiconductor surfaces,^143–146^ should provide an exciting opportunity to obtain chemical control over the properties of these semiconductor surfaces. Oxides have been functionalized extensively for use in dye-sensitized solar cells,^147–149^ and a similarly favorable outlook for progress in defining the chemistry of surface states and surface sites is applicable to binary oxides such as TiO_2_ and ternary oxides such as BiVO_4_.^150–155^ Surfaces that have a mixture of oxides at the interface are more complicated to define and understand based on their reactivity, but progress is evident on such systems as well. For example, the reactivity of GaAs with a series of substitution inert and substitution labile transition-metal complexes^156^ could be correlated to the surface recombination velocity of the modified interfaces and the bonding characterized spectroscopically using a variety of operando methods including ambient pressure X-ray photoelectron spectroscopy.^157^

Another frontier area involves understanding how to manipulate the band-edge positions from a molecular perspective, often denoted as “band-edge engineering”.^158–161^ Figure 6a displays the band bending associated with an equilibrated n-type semiconductor–liquid contact. The introduction of a strongly adsorbed charged species to this interface forms a surface dipole, which shifts the position of the band-edges and thus alters the magnitude of band bending (Figure 6b).^42,162,163^ The approach should be broadly applicable to a variety of semiconductor electrode materials, provided that the surface bonding and composition is well-defined and well-elucidated.^164^ For example, the band-edge position of Si surfaces can be rationally manipulated by covalent attachment of organic species through formation of Si–C bonds on the H-terminated Si(111) surface.^44,162,165,166^ Alkyl groups introduce a surface dipole that shifts the band edges toward the vacuum level, whereas introduction of electron withdrawing groups shifts the band edges away from the vacuum level.^166–171^ Molecular-level control over the band-edge positions would provide systematic increases in the photovoltage, and efficiency, of either n-type Si photoanodes or p-type Si photocathodes in contact with a variety of electrolytes, without introduction of deleterious surface trap sites that degrade the photoelectrode performance.

**Figure 6:**
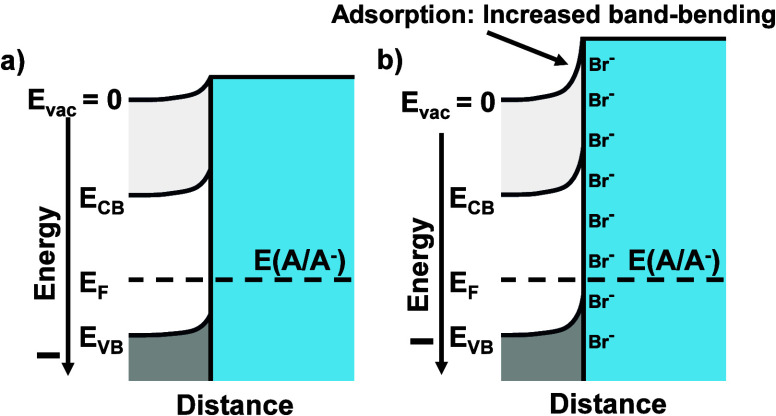
Energy-level diagrams displaying the shift in band-edge positions due to adsorption of negatively charged species onto an n-type semiconductor–liquid contact. (a) To establish charge-transfer equilibrium and equilibrate the Fermi level (i.e., the electrochemical potential) throughout the system, charge flows from the solid into the liquid, leaving the solid with a net positive charge and the liquid having excess negative charge. The negative charge (not shown) in the liquid is in the compact double layer (Helmholtz layer) of the electrolyte. The positive charge (not shown) results in a depletion region in the semiconductor. Band bending is produced in the solid because work is required to move a test point negative charge from the bulk of the solid through the depletion region to the sheet of negative charge located at the solid–liquid interface. In (b), when additional negative charge is adsorbed onto the semiconductor surface, more band bending is produced, reflecting the increased difficulty of moving a test point charge through the expanded depletion region to the solid–liquid interface. This situation physically is equivalent to moving the band-edge positions closer to the vacuum level, i.e., shifting the band-edge positions of the semiconductor–liquid interface, in a process called “band-edge engineering”.

Building on these advances, the outlook is favorable for obtaining unprecedented control over the band-edge positions of other semiconductor photoelectrodes.

Surface functionalization reactions associated with adsorption of chalcogens, halogens, or organic species on the polar (111) face of other II–VI semiconductor materials such as CdS, CdSe, CdTe, and ZnO, can be expected to provide analogous molecular-level control of the band-edge positions at solid–liquid interfaces based on these semiconductors.^172–178^

Arguably the biggest challenge in semiconductor photoelectrochemistry is electrode durability, especially for photoelectrodes that are performing the oxygen-evolution reaction in water or effecting fuel-forming reduction half reactions like H_2_ production and/or CO_2_ reduction.^88,106,108^ The problem of stability has been at the forefront of the field for over 50 years, with the fundamentals elucidated theoretically and experimentally by Gerischer beginning in the 1960s.^25,179,180^

Stability under fuel-forming conditions is especially challenging because intrinsically safe operation of a practical solar-based water splitting system requires separate streams of H_2_(g) and O_2_(g), with gas concentrations below the lower explosive limit at all positions and times in the system.^106,181–186^ Additionally, maintaining a constant composition of the catholyte and anolyte over time requires a transference number of unity for protons across the membrane in the electrolytic cell, and consequently requires exclusively either protons moving in the same direction as the electrons, and/or of hydroxide moving in the opposite direction as the electrons.^187–190^ Thus, photoelectrodes must operate at either extremely low or extremely high pH conditions to ensure the safe, efficient, steady-state production of solar fuels.^182,183,191^ Otherwise, a pH gradient will be produced in the cell, with no mechanism to beneficially use the resulting proton-motive force except to dissipate the pH gradient unproductively by mixing the anolyte with the catholyte to restore the cell to its initial chemical state.^183,185^ Membrane-free systems have been explored in the laboratory^192^ but the stability of the flow under variable current densities, operating temperatures and diurnal cycling is an unresolved issue.

Theoretical and experimental Pourbaix diagrams are now available for many semiconductor electrodes.^193–197^ In combination with surface analysis methods and product analysis methods, the primary failure modes under various operating conditions of semiconductor electrodes can be definitively identified and then systematically mitigated. Photoanodes are unstable under such oxidative operating conditions either due to passivation or due to pitting corrosion by dissolution.^25,198,199^ Protection layers, often based on TiO_2_, have been developed to allow holes to conduct through thick layers while preventing direct contact between the liquid and the electrode, thus minimizing corrosion.^200–206^

Some semiconductors, such as silicon, form a passivating oxide over a wide pH range.^193,207^ When coated with a protection layer, photoanodes that are operated in the passivation regime can exhibit extended stability for thousands of hours of continuous operation under simulated sunlight.^208^ Pinholes in the protection layer then only lead to formation of a passivating oxide layer while the remainder of the photoanode remains intact and functional.^199^ Methods of stabilizing such photoanodes have even been recently developed to deal with the different corrosion processes associated with photoelectrode operation under dark versus light conditions associated with the diurnal insolation cycle.^209,210^ Although Si is anodically passivated in 1.0 M KOH­(aq) under illumination, Si undergoes dissolution in the dark at open-circuit in such electrolytes.^199,211^ This dissolution can however be suppressed by addition of a recyclable reagent, or even by a water oxidation catalyst (such as Ni oxyhydroxide), that maintains the photoanode at its passivation potential, or more positive than its passivation potential, in the dark as well as under illumination.^199^

Such approaches provide a favorable outlook for obtaining step-changes in the stability of a variety of heretofore unstable photoanode materials and systems.

Other semiconductors, such as the III–V materials based on alloys of GaAs, InP, and/or GaP, undergo corrosion by oxidative passivation at near-neutral pH, but undergo corrosion by dissolution of soluble oxides in either acidic or alkaline media.^25,212–215^ Pitting corrosion can spread throughout the substrate eventually leading to catastrophic failure of the entire electrode.^198,199,216^ Through a combination of theory, experiment, and surface science methods, the corrosion process has been identified and resolved spatially to occur at pinhole defect sites in the oxide protection layer. Consequently, an apparently general mitigation strategy has been recently developed involving the use of microwire or microisland structures that are not in mutual electrical contact (Figure 7a,b). Only a small percentage of the regions will in practice have a pinhole as a result of deposition of the protection layer under atmospheric ambient conditions.^217^ In contrast with phenomenological measurements of photocurrent stability,^202^ applying these methods provides a systematic, rational approach to extending the stability to other semiconductor photoanodes that undergo corrosion at defects in protection layers.

**Figure 7:**
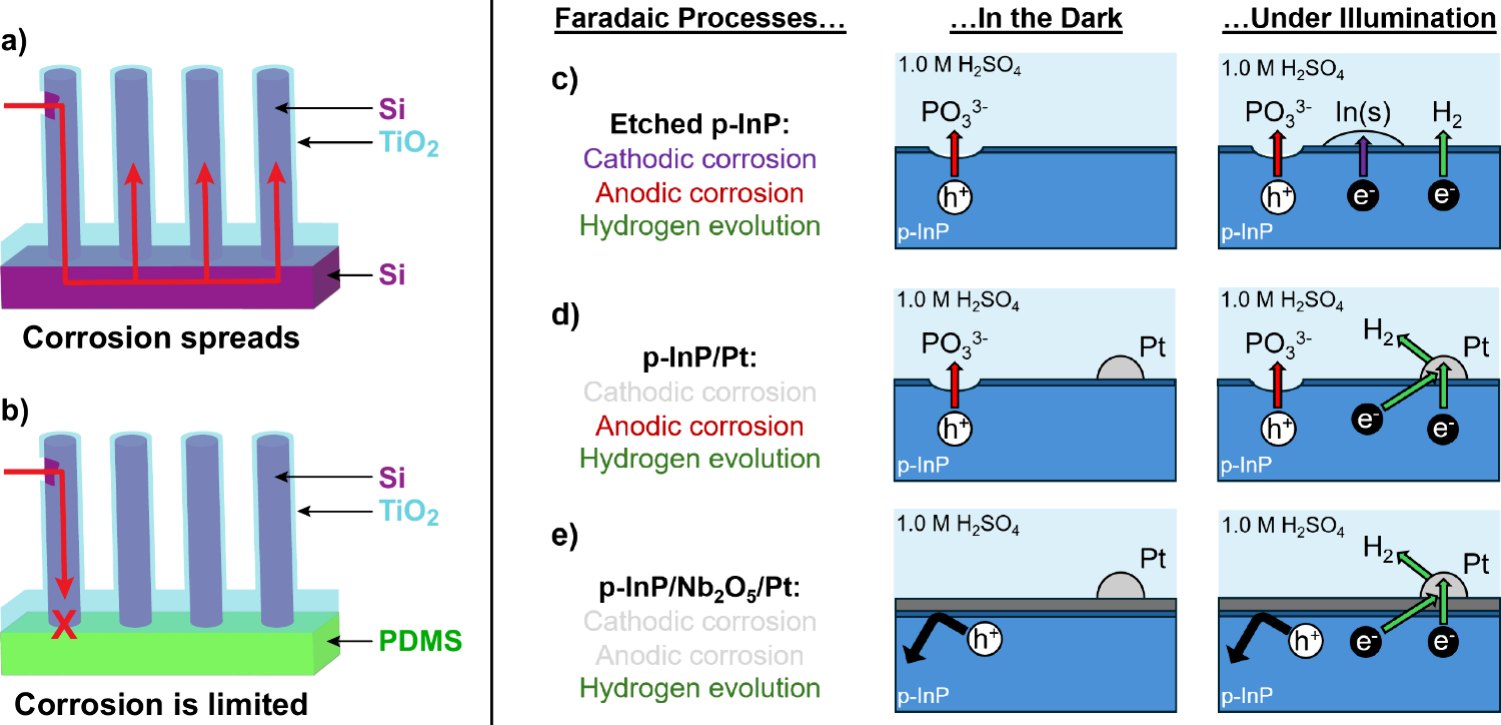
Schematic of different types of physical and chemical corrosion processes at a semiconductor photoelectrode. (a) An array of Si microwires is grown on a Si substrate. The microwires are coated with a TiO_2_ protection layer. Under conditions where the Si corrodes by dissolution, the corrosion initiates at pinholes in the protection layer and spreads through the substrate to all of the wires, eventually leaving no remaining Si. (b) The TiO_2_-protected microwire array has been removed from the Si substrate and embedded into an insulating (polydimethylsilane, PDMS) polymer membrane. Only the small fraction of microwires that have a pinhole corrode by dissolution, and the remainder remain intact. (c) Chemical corrosion pathways of an etched p-InP photocathode. Without illumination, corrosion occurs at unprotected regions of the material by chemical and/or anodic dissolution. With illumination, without Pt, the photogenerated electrons reduce the InP and plate In metal. (d) Corrosion pathways of platinized p-InP in acidic media with and without, respectively, illumination. Without illumination, platinized p-InP corrodes in the same manner as etched p-InP. With illumination, anodic corrosion continues but cathodic corrosion due to In plating is suppressed in favor of hydrogen evolution. (e) Corrosion processes of Nb_2_O_5_-coated p-InP with a Pt catalyst with and without, respectively, illumination. In both cases, both the cathodic and anodic corrosion processes are suppressed. Under illumination, the HER proceeds without the detrimental corrosion processes.

Either under illumination or in the dark, photocathodes undergo both chemical and electrochemical anodic corrosion at positive potentials near open-circuit (Figure 7c,d).^213,214,216^ While under illumination, photocathodes are generally cathodically protected from corrosion resulting from plating of metal (Figure 7d).^180,193,214^ The availability of theoretical and experimental Pourbaix diagrams from the Materials Project also provides insight into rational approaches to development of protection layers for photocathodes. For example, TiO_2_, SnO_2_, and other oxides have been widely used as protection layers for photocathodes performing the HER in acidic media.^202^ Notably, the Pourbaix diagrams indicate that all of these oxides are thermodynamically unstable in acidic media at the hydrogen evolution potential. Stimulated by these findings, recent experiments have shown that these oxides rapidly dissolve under such conditions, illustrating the confounding effects of relying solely on phenomenological measurements of photocurrent stability for photocathodes that are kinetically stabilized by suitable electrocatalysts decorated on the electrode surface in the absence of any protection layers.^218^ Nb_2_O_5_ is however predicted to be stable at the hydrogen-evolution reaction potential in acidic aqueous solutions. Consistently, Nb_2_O_5_ films deposited by atomic layer deposition were recently shown to provide a chemically and electrochemically stable protection layer in acidic media for platinized p-InP photocathodes performing the hydrogen evolution reaction (Figure 7e). Nb_2_O_5_ layers formed by atomic layer deposition moreover suppress the primary failure mode of anodic and/or chemical dissolution of the p-InP in the dark and/or at very positive electrode potentials.^218^ The photovoltage of Nb_2_O_5_-protected p-InP/Pt photocathodes is ∼200 mV less than the photovoltage of etched p-InP/Pt for hydrogen evolution, but improvements should be possible through implementing a chemical functionalization approach to band-edge engineering. Hence, through developing the fundamental science of protection layers for photocathodes, involving overlayers that do not impede flow of minority carriers (i.e., electrons for photocathodes and holes for photoanodes) while still blocking the chemical and electrochemical corrosion processes that limit the durability of these materials when operated under diurnal insolation cycles, the outlook is favorable for step-changes in photocathode stability and performance under a variety of operating conditions.^219–221^ Understanding and effectively mitigating semiconductor corrosion and instability is still of paramount importance to enabling practical implementation of semiconductor photoelectrodes as a viable energy-conversion technology.^222–224^

Durability considerations of solar fuels systems are not limited only to photoelectrodes. Most semiconductor surfaces are not inherently active catalysts for the multielectron transfer half-reactions associated with water oxidation or for the reduction of water and/or CO_2_.^89,90,225,226^ Electrocatalysts that have C–H bonds are unlikely to be stable under oxidative conditions, especially in the presence of traces of hydroxyl radicals in the multielectron oxidation of two water molecules to form one O_2_ molecule. Similarly, electrocatalysts that have organic unsaturation are unlikely to be stable under reductive conditions to form H_2_, and will be susceptible to hydrogenation and/or to attack by traces of superoxide produced by reduction of O_2_ that has crossed over from the anolyte into the catholyte. Durable systems will likely require electrocatalysts that are devoid of these reactive groups, to facilitate fuel forming half-reactions with practically relevant lifetimes and turnover numbers under actual operating conditions.^227–229^ These considerations are also applicable to emerging and proposed molecular-based systems for solar fuels production, including the light absorbers, catalysts, and separators of such constructs.

A new generation of earth-abundant electrocatalysts for the hydrogen-evolution reaction in acidic media has been identified using this approach. Ni_2_P is a thermal catalyst for hydrodesulfurization, proceeding through a metal-hydride intermediate formed by cleavage of dihydrogen. Also, a select crystal face of Ni_2_P was theoretically predicted to provide an active electrochemical hydrogen evolution catalyst by proceeding through an analogous metal hydride intermediate.^230^ Consequently, in conjunction with recognizing the high catalytic activity exhibited by transition metal NiP_2_N_2_ complexes for hydrogen evolution, nanoparticles of Ni_2_P were shown to provide a low overpotential, earth abundant, relatively stable HER catalyst in acidic media.^227^ Subsequently, the entire series of first row transition metal phosphides was explored, as well as nanostructured metal phosphides and sulfides, to produce even more active and stable electrocatalysts under operating conditions.^227,228,231–245^ These electrocatalysts have different interfacial chemistry with the underlying semiconductor than Pt or noble metal electrocatalysts, and should allow for unique performance when integrated with photoelectrodes because the transition metal phosphides and sulfides do not subject the semiconductor electrode to the same galvanic displacement processes that induce corrosion and/or interfacial oxide-formation that are produced when Pt, Au, or Ir are deposited onto such electrode surfaces.

Considering the global frenzy of activity associated with optimizing these electrocatalysts, the outlook is very favorable for exploiting the tunable hydricity of these systems to effect numerous important multielectron transfer reduction processes, including CO_2_ and N_2_ reduction in addition to H_2_ evolution.

^231–245^

The development of stable, efficient photoelectrochemical cells is necessary, but not sufficient, to allow semiconductor photoelectrodes to compete technologically with a separate, discrete photovoltaic system connected to an electrolyzer for solar fuels production. Protection layers have enabled demonstration of water-splitting systems,^181,246^ and of highly selective CO_2_ reduction systems for formate production,^247^ at the laboratory scale for several days of operation with greater than 10% solar-to-fuels energy-conversion efficiency. Also, metal–insulator–semiconductor photocathodes have demonstrated moderate selectivity of CO_2_ reduction to ethylene for several hours.^248^ However, even if grid-connected electrolyzers that use electricity produced by photovoltaics have nominally identical efficiencies and durability as an integrated photoelectrochemical solar fuels system, grid-connected electrolyzers can also use wind, solar, and nuclear electricity. Consequently, grid-connected electrolyzers can operate at >80% capacity factors with carbon-free electricity derived solely from variable renewables,^249,250^ allowing for cost-effective utilization of capital and minimizing the balance of systems cost associated with collection of hydrogen at low pressures over relatively large areas.^251,252^ Hence, technoeconomic analysis indicates that other potential advantages of photoelectrochemical systems must be exploited to obtain a system that is cost-competitive with grid-connected electrolysis for fuel formation.^108^ Such advantages could involve exploitation of approaches that are uniquely facilitated by semiconductor–liquid interfaces, to allow high energy-conversion efficiency from very inexpensive light absorbers and catalyst materials. For example, the light absorbers in dye-sensitized solar cells are readily manufactured inexpensively at scale and can provide energy-conversion efficiencies above 15%.^253^ However, dye-sensitized solar cells have not yet exhibited the durability required for wide-scale commercial implementation for production of solar electricity.^254,255^ Also, at present, solar fuels systems based on dye-sensitization have low energy-conversion efficiencies and are not intrinsically safe in operation because they produce unseparated, stoichiometric ratios of products in the presence of active catalysts for chemically-based recombination.^256–258^ Light absorbers based on small grain polycrystalline semiconductors with passivated grain boundaries could provide an opportunity to obtain high efficiency at a solid–liquid contact. Examples include polycrystalline CdTe “solar paint” or use of polycrystalline p-InP treated with Ag ions to passivate grain boundaries,^109,112,259–261^ exploiting the liquid electrolyte as the front contact to transport only minority carriers via the redox-active species in the liquid phase, minimizing majority carrier shunting (Figure 8a). In contrast, a metal front contact allows a large majority carrier current, producing complete front-surface recombination (Figure 8b).

Surface functionalization and capping nanoparticles during synthesis with electronically conductive linkers coupled to either the semiconductor conduction band or valence band could thus provide a novel, promising approach to synthesis of materials that simultaneously exhibit the high mobility and long charge-carrier lifetime required to make efficient photoelectrodes from small grain size, high surface area nanoparticles.

Furthermore, structure–function relations can be obtained using photoluminescence, single-molecule and single-particle methods, and scanning probe techniques to characterize these systems in unprecedented detail.

**Figure 8:**
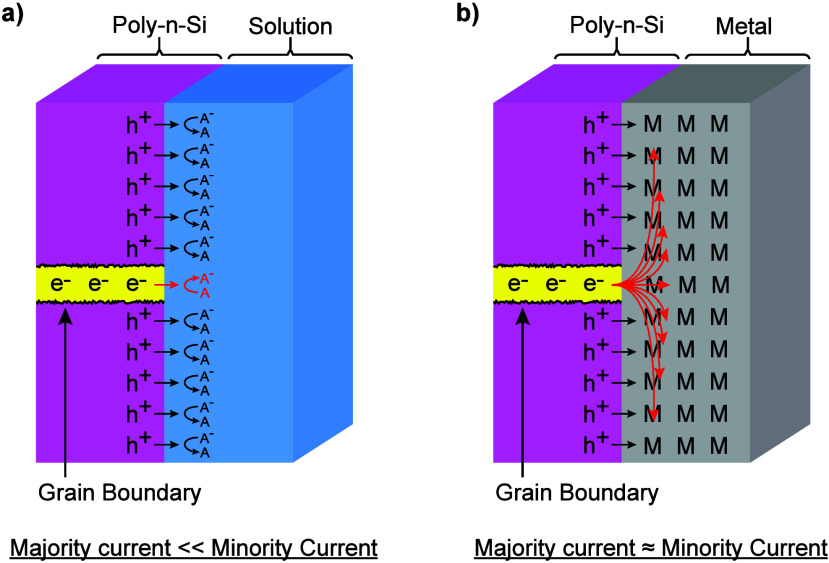
Majority- and minority-carrier transport in polycrystalline n-Si with (a) a liquid front contact and (b) a metal front contact. For the liquid-contacted electrode (a), majority carriers can only reach the interface through the grain boundaries. Because the grain boundary area in contact with the electrolyte is small, the resulting majority-carrier current is limited by the mass transport of redox-active species in solution. In contrast, the majority-carrier current in the metal-contacted electrode (b) can match the minority-carrier current, despite the difference in contact areas for the two carrier types, producing nearly complete front-surface recombination and preventing a net photocurrent from flowing in the external circuit.

Another opportunity involves exploiting novel morphologies at semiconductor–liquid contacts to uniquely allow high efficiency from low-cost materials. Nanowire and microwire arrays provide examples of the approach, in which the high aspect ratio of the structured electrode surface provides a long axis to facilitate high absorption of light, with the short axis allowing for efficient collection of minority carriers at the conformal solid–liquid front contact (Figure 9).^262–265^ This approach also allows for optimal placement of catalysts to minimize deleterious optical and mass transport effects associated with evolution of gas bubbles at the photoelectrode surface, while also allowing for minimization of optical reflection and absorption losses commonly associated with the use of thick catalyst layers required to minimize overpotentials for fuel formation at the photoelectrode surface.^262,266–269^

**Figure 9:**
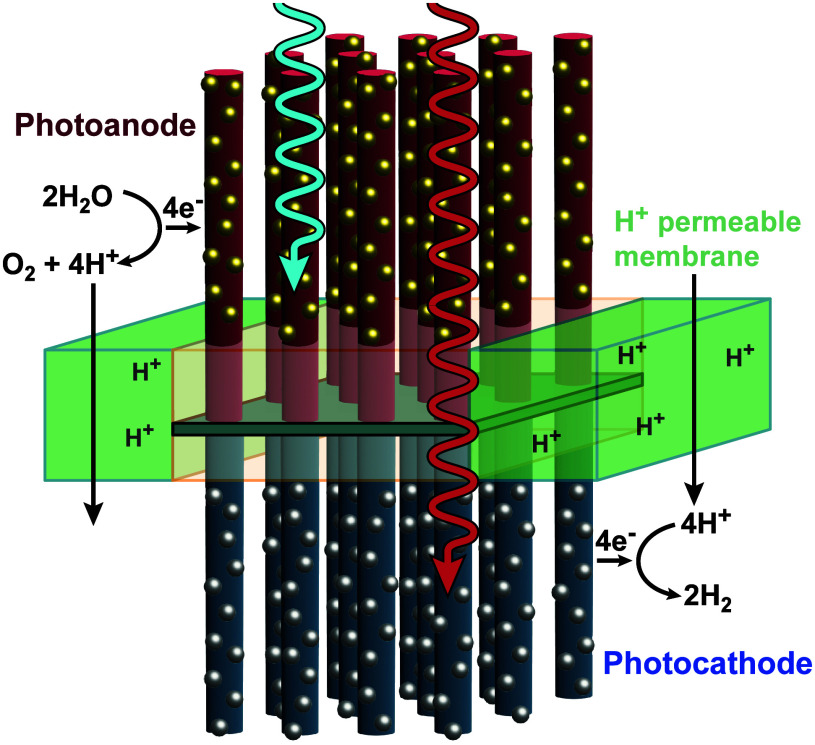
Schematic of a water-splitting device utilizing dual, structured solar absorbers and a proton-permeable membrane for ion transport. The high-aspect-ratio structures provide effective light absorption at a variety of angles of incidence. Orthogonalization of the directions of light absorption and carrier-collection enables efficient use of low-purity semiconductor materials with short collection lengths for photoexcited charge carriers. The controlled porosity allows for optimization of reactant access and product egress, and optimization of catalyst loading and placement to maximize product formation while minimizing optical obscuration of the light absorbers due to metallic catalyst particles. The surface texture of the microstructure allows for control over the size and transport of H_2_ and O_2_ bubbles under illumination, thus maximizing the contact area between the liquid and the solid, while allowing for facile gaseous product evolution even against gravity. The membrane provides mechanical support, separation of the product gases, transport of protons and/or hydroxide to maintain electroneutrality in the cell, and electrically isolates each microwire from the remainder of the array to prevent spreading of corrosion sites on a few microwires from leading to dissolution of the entire array of semiconductor material. Reproduced from ref 262. Copyright 2013 American Chemical Society.

Integrated photoelectrochemical systems for production of solar fuels could also beneficially exploit the potentially unique catalytic activity of semiconductor surfaces, such as methanol formation on GaAs,^270^ which is not readily effected on the metal electrode surfaces and metal electrocatalysts that would be present in grid-connected electrolyzers.

Another opportunity is associated with the use of semiconductor electrochemistry to produce unique mesostructures in materials. Photoelectrochemical etching is a well-developed technology, relying on corrosion produced by spatially patterned photoexcitation of a semiconductor electrode to form microlenses and other structures on the semiconductor surface.^271–273^ In contrast, inorganic phototropic growth by light-stimulated electrodeposition of p-type semiconductors involves growth of material toward the direction of an incoherent, unstructured, low-intensity source, such as a light emitting diode, an incandescent bulb, or the sun. The morphology is further defined by the spectral distribution, polarization, intensity, propagation direction, relative phase, etc. of the illumination during growth.^274–277^ Growth in the dark results in an isotropic deposit without substantial long-range order (Figure 10a). Growth under unpolarized illumination results in a nanopore array (Figure 10b), whereas growth using a linearly polarized optical input generates an anisotropic lamellar array. The long axes of the lamellae are oriented along the input polarization (E-field) vector and the spacing between the lamellae, i.e., the feature pitch, can be set arbitrarily by defining the spectral distribution of the illumination (Figure 10c–e). The out-of-plane feature orientation is controlled by the propagation direction of the stimulating light beam relative to the substrate. For example, features grow normal to the substrate under illumination with normal incidence, whereas inclined illumination results in growth of inclined features, i.e., growth toward the light source (Figure 10f). Moreover, the deposit morphology is a function of all simultaneous illumination inputs during growth, e.g., two orthogonally polarized inputs can generate intersecting structures (Figure 11a–c).^275^ Additionally, time-varying optical inputs can generate three-dimensional intricacy. For example, abrupt changes in the input wavelength result in the growth of aqueduct and tuning fork structures (Figure 11d–f).^278–280^

**Figure 10:**
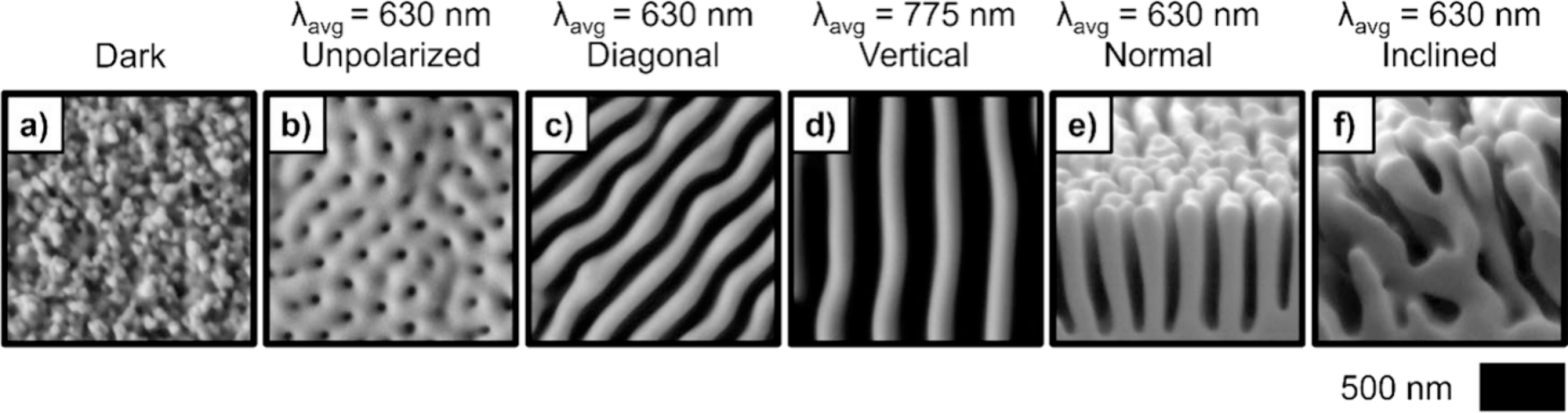
(a–d) Top-down and (e, f) cross-sectional scanning electron micrographs (SEMs) of Se–Te deposits generated via inorganic phototropic growth. The 3D mesostructures result from emergent light–matter interactions during the photoelectrodeposition of p-type semiconductors, and are produced despite the use of spatially uniform, incoherent, low intensity illumination, and optically isotropic electrodes and solutions. Adapted from ref 281.

**Figure 11:**
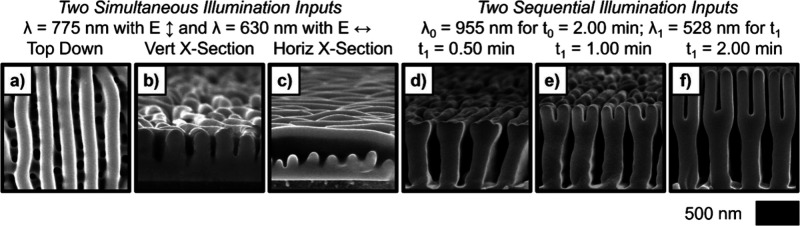
SEMs of Se–Te deposits generated (a–c) using simultaneous vertically polarized λ = 775 nm and horizontally polarized λ = 630 nm illumination and (d–f) first using λ = 955 nm illumination and then extended with a sequential growth step with λ = 528 nm illumination for the indicated duration. The 3D morphologies of these photoelectrochemically deposited materials are completely controlled by the wavelength, intensity, and polarization of the unstructured, incoherent, illumination sources used to stimulate the deposition process. (a–c) Adapted from ref 275. Copyright 2015 American Chemical Society. (d–f) Adapted from ref 278. CC BY-NC-ND 4.0.

Consequently, inorganic phototropic growth represents a fundamentally new, transformational way to deposit materials, control nanoscale morphology, and produce complex 3D structures in a single step, with no identified barriers to scale.

As a result, inorganic phototropic growth represents an emerging area of semiconductor photoelectrochemistry, with potential applications in controlling the wetting, hydrodynamic flow, porosity, and optoelectronic properties of multifunctional materials and surface coatings.^274–277,279,281–285^

In summary, tremendous progress has been made in understanding the fundamentals of semiconductor photoelectrochemistry and in validating the basic theoretical understanding of interfacial charge-transfer at semiconductor electrode surfaces. Applications to energy conversion will require solving persistent, thorny issues associated with multielectron transfer processes, electrode stability under stressing operational conditions including day/night cycling, and exploiting unique chemical reactivity and advantageous charge-carrier generation and collection characteristics of mesostructured semiconductor electrodes, to develop practical, efficient, safe, durable, and cost-effective systems for solar fuels generation. Other research opportunities abound for semiconductor photoelectrodes including fundamental aspects of complex surface chemical reactions and surface passivation, understanding both fundamental and use-inspired aspects of inner-sphere electron-transfer processes, and exploiting novel applications of light-stimulated etching and morphology control, ensuring ample challenges in semiconductor photoelectrochemistry for many years in the future.
